# Human Hepatic CD56^bright^ NK Cells Display a Tissue-Resident Transcriptional Profile and Enhanced Ability to Kill Allogenic CD8^+^ T Cells

**DOI:** 10.3389/fimmu.2022.921212

**Published:** 2022-07-05

**Authors:** Gráinne Jameson, Cathal Harmon, Rhyla Mae Santiago, Diarmaid D. Houlihan, Tom K. Gallagher, Lydia Lynch, Mark W. Robinson, Cliona O’Farrelly

**Affiliations:** ^1^ School of Medicine, Trinity Translational Medicine Institute, Trinity College Dublin, Dublin, Ireland; ^2^ Brigham and Women’s Hospital, Harvard Medical School, Boston, MA, United States; ^3^ Department of Biology, Kathleen Lonsdale Institute of Human Health Research, Maynooth University, Maynooth, Ireland; ^4^ Liver Unit, St. Vincent’s University Hospital, Dublin, Ireland; ^5^ Hepatopancreaticobiliary Group, St. Vincent’s University Hospital, Dublin, Ireland; ^6^ School of Biochemistry and Immunology, Trinity Biomedical Sciences Institute, Trinity College Dublin, Dublin, Ireland

**Keywords:** NK cell, resident, CD8+ T cell, liver, transplant, tolerance

## Abstract

Liver-resident CD56^bright^CD16^-^ natural killer (NK) cells are enriched in the human liver and are phenotypically distinct from their blood counterparts. Although these cells are capable of rapid cytotoxic effector activity, their functional role remains unclear. We hypothesise that they may contribute to immune tolerance in the liver during transplantation. RNA sequencing was carried out on FACS sorted NK cell subpopulations from liver perfusates (n=5) and healthy blood controls (n=5). Liver-resident CD56^bright^CD16^+/-^ NK cells upregulate genes associated with tissue residency. They also upregulate expression of *CD160* and *LY9*, both of which encode immune receptors capable of activating NK cells. Co-expression of CD160 and Ly9 on liver-resident NK cells was validated using flow cytometry. Hepatic NK cell cytotoxicity against allogenic T cells was tested using an *in vitro* co-culture system of liver perfusate-derived NK cells and blood T cells (n=10-13). In co-culture experiments, hepatic NK cells but not blood NK cells induced significant allogenic T cell death (p=0.0306). Allogenic CD8^+^ T cells were more susceptible to hepatic NK cytotoxicity than CD4^+^ T cells (p<0.0001). Stimulation of hepatic CD56^bright^ NK cells with an anti-CD160 agonist mAb enhanced this cytotoxic response (p=0.0382). Our results highlight a role for donor liver NK cells in regulating allogenic CD8^+^ T cell activation, which may be important in controlling recipient CD8^+^ T cell-mediated rejection post liver-transplant.

## Introduction

The human liver is populated with a unique repertoire of immune cells, many of which are distinct from their circulating counterparts (1, 2). The hepatic immune repertoire includes enriched innate lymphoid populations such as NK cells, γδ T cells (3, 4), invariant NKT cells (5, 6) and MAIT cells (7, 8), as well as adaptive CD8^+^ T cells (2). These hepatic immune cell populations need to remain tolerant to the continuous exposure to harmless dietary and commensal bacterial products arriving from the gut *via* the portal vein, while simultaneously being ready to mount an immune response upon insult by infectious agents or malignant cells (9).

NK cells constitute on average 50% of the total liver lymphocyte population and include both circulating and liver-resident cells (10). Human liver-resident NK cells are CD56^bright^ and are phenotypically distinct from circulating NK cell populations (10–13). They express the chemokine receptor CXCR6, the C-type lectin CD69, lack the integrin CD49e (ITGA5) (13, 14), and display different transcription factor usage, with high levels of eomesodermin (Eomes) and low levels of T-box transcription factor TBX21 (T-bet) (10). These liver-resident cells are also long-lived and/or capable of self-renewal in humans, having been found up to 13 years post-liver transplantation in transplant recipients who received HLA-mismatched donor livers (15). At a functional level, liver-resident NK cells display reduced cytokine responses and increased cytotoxicity against tumour cell lines in comparison to their peripheral blood counterparts (10, 11, 14, 16–18). In the context of chronic hepatitis-B virus (HBV) infection liver-resident NK cells up-regulate expression of TRAIL and possess the ability to suppress HBV-specific T cell populations (19, 20). While TRAIL is expressed in disease, it is absent on liver-resident NK cells in healthy liver (21). It is unknown whether liver-resident NK cells maintain the ability to suppress T cell function, independent of TRAIL, as part of the tolerogenic nature of the liver.

Liver transplantation is influenced by the tolerogenic immune environment of the liver (22, 23). In comparison to recipients of other solid organ transplants, liver transplant recipients are not HLA-matched, require less immunosuppression, have a lower incidence of acute and chronic rejection and a proportion of transplant recipients can be weaned off immunosuppression without organ rejection (24–28). We propose that liver NK cells play a role in inducing and maintaining liver tolerance. Post-liver transplant, recipient T cells are activated by the recognition of donor alloantigen (29) resulting in their infiltration into the liver where they can mediate tissue damage and may lead to allograft rejection (30). Rapid decreases in donor specific CD8^+^ T cells are observed in liver grafts recipients with good graft function, indicative of T cell inactivation and the development of tolerance (31). The importance of donor leukocytes in mediating tolerance is evidenced by studies showing that irradiation of rodent liver allografts prior to transplantation resulted in their rapid rejection (32, 33), which could be reversed by the replenishment of donor leukocyte populations (34). Understanding the mechanisms underlying the induction of tolerance is important in elucidating strategies to promote allograft acceptance post-transplant and reduce the need for immunosuppressive drugs.

In this study, we performed transcriptomic analysis of human hepatic and circulating NK cell populations and analysed this data for differentially expressed genes that may be related to regulatory functions specific to liver-resident NK cell populations. We validated these gene expression differences at the protein level and performed *in vitro* co-cultures of liver NK cells with allogenic T cells. Our results highlight a potential role for liver-resident NK cells in liver tolerance post-liver transplant via the regulation of allogenic activated CD8^+^ T cells.

## Methods

### Collection of Liver and Peripheral Blood Samples

Liver samples were collected from donor livers during orthotopic liver transplantation at St. Vincent’s University Hospital. During retrieval, the donor aorta and superior mesenteric vein were flushed with University of Wisconsin solution (Bristol-Myers Squibb, Uxbridge, UK) at the time of exsanguination. The liver was flushed again after excision of the organ until all blood was removed and the perfusate appeared clear, at which time the liver was placed in a container with University of Wisconsin solution and packed on ice for transportation. Donor livers were transplanted within 12 hr. At implantation, after completion of the upper inferior vena cava anastomosis, livers were flushed through the portal vein before reperfusion. This wash-out fluid was collected from the inferior vena cava, along with the transportation media. Peripheral blood was obtained from anonymized blood donors from the Irish Blood transfusion Board. All protocols were approved by St. Vincent’s University Hospital Ethics Committee in accordance with the ethical guidelines of the 1975 Declaration of Helsinki.

### Isolation of Hepatic Mononuclear Cells (HMNC) and Peripheral Blood Mononuclear Cells (PBMC)

Hepatic mononuclear cells (HMNC) were isolated from healthy tissue samples by mechanical digestion with a scalpel followed by enzymatic digestion (Miltenyi Biotec, Bergisch Gladbach, Germany) for 20 min with shaking at 180 rpm at 37°C. Full details on the reagents and kits used are provided in **Supplementary Table 1**. Digested tissue was then filtered through 70 μm filters (BD Biosciences, Erembodegem, Belgium) with phosphate buffered saline and centrifuged at 30 rcf for 3 min to pellet and remove hepatocytes. The supernatant was centrifuged at 300 rcf for 5 min to pellet the cells and then resuspended in complete RPMI (RPMI 1640 medium supplemented with 10% fetal calf serum (FCS) and 1% penicillin/streptomycin (Gibco, Paisley, UK)). HMNCs were isolated from liver perfusate by filtration through 70 μm filters followed by centrifugation at 600 rcf for 10 min. The supernatant was aspirated and pelleted cells were resuspended complete RPMI.

Both tissue-derived and perfusate-derived HMNCs were isolated by density gradient centrifugation using Ficoll-Paque™ PLUS (GE Healthcare, Uppsala, Sweden) and a red blood cell lysis was performed. For the red blood cell lysis a 10x stock solution was prepared by dissolving 8.02g NH_4_Cl (ACROS Organics, New Jersey, USA) and 1g KHCO_3_ (ACROS Organics) in 100mL of ddH_2_O, then adding 0.2 mL EDTA 0.5M (Invitrogen, California, USA). A 1x working solution was diluted in ddH_2_O and filter sterilised immediately prior to use. Cells were then stained for analysis by flow cytometry. Non-matched anonymous peripheral mononuclear cells (PBMC) were also isolated by density centrifugation using Ficoll-Paque™ PLUS. Both perfusate-derived HMNC and PBMC samples were cryopreserved in liquid nitrogen in a 90% FCS and 10% DMSO medium (Fisher BioReagents, Pennsylvania, USA).

### NK Cell Isolation for RNA Sequencing

For the isolation of CD56^bright^ and CD56^dim^ NK cell subpopulations cells were stained with the following antibodies: CD56, clone NCAM16.2; CD16, clone 3G8; CD3, clone UCHT1; CD45, clone HI30 (all BD Biosciences); CD127, clone REA614 (Miltenyi Biotec); with propidium iodide (ThermoFischer Scientific, Massachusetts, USA) for live/dead discrimination. Full details of the antibody panels used for flow cytometry are provided in **Supplementary Table 2**. NK cell subpopulations were then sorted to >98% purity using either a FACS Aria or a FACS Melody (BD Biosciences) using the gating strategy defined in **Supplementary Figure 1**. Sort purity was assessed to be >98% by re-running the sorted populations.

### RNA Sequencing

A total of 1,500 cells of each sub-population of interest was FACS sorted into 1x TCL buffer (Qiagen, Manchester, UK) with 1% b-mercaptoethanol (molecular biology grade, v/v; Sigma-Aldrich, Missouri, USA). Sorted samples were frozen on dry-ice and stored at -80°C until library preparation. Full-length transcripts were sequenced from low-input samples by the Broad Institute using a modified version of the SMART-Seq2 (35, 36). The generated libraries were sequenced on an Illumina NextSeq500 using a High Output kit to generate 2 x 25 bp indexed reads. Sequencing data quality was assessed using FastQC, reads were aligned using HISAT2 onto the UCSC hg38 genome build (37), and multi-mapping reads were removed from the BAM files (**Supplementary Figure 2**). Normalisation via the TMM methods and differential expression analysis was performed on Qlucore Omics Explorer 3.2 and subsequent visualisation was performed in R. Sequencing data files are accessible through NCBI’s Gene Expression Omnibus Series accession number GSE200319 (https://www.ncbi.nlm.nih.gov/geo/query/acc.cgi?acc=GSE200319).

### NK and T Cell Cocultures

Frozen HMNCs were recovered and rested in complete RPMI supplemented with 2ng/mL IL-15 (Miltenyl Biotec) and 25ug/mL DNAse-I (Sigma-Aldrich) for 45 mins at 37°C in 5% CO_2_. CD56^+^ NK cells were magnetically isolated from HMNCs using a negative selection kit (Stemcell, Vancouver, Canada) and from peripheral blood samples using a RosetteSep™ kit (Stemcell). Whole PBMCs were isolated by density centrifugation using Ficoll-Paque™ PLUS and CD3^+^ T cells were magnetically isolated from PBMCs using a negative selection kit (Stemcell). PBMCs or CD3^+^ T Cells were stimulated with anti-CD3 (5µg/mL; Tonbo Biosciences, California, USA) and anti-CD28 (2µg/mL; Tonbo Biosciences) and co-cultured with either hepatic or peripheral blood NK cells at a 1:1 (PBMC : NK) ratio in cRPMI supplemented with 2ng/mL IL-15, unless otherwise indicated. A monoclonal antibody (mAb) against CD160 (MBL, Massachusetts, USA) or an IgG isotype control (Biolegend, California, USA) was added at 10µg/mL to assess its role in NK cell killing. After 24 hr the percentage of dead T cells was assessed using the coculture flow cytometry panel (**Supplementary Table 2**).

### Flow Cytometry Analysis

Cells were transferred to 5mL flow tubes and centrifuged at 300 rcf for 5 min in phosphate buffered saline supplemented with 1% bovine serum albumin (GE Healthcare, Uppsala, Sweden) and 1mM EDTA (ThermoFischer Scientific). Supernatant was aspirated and cells were resuspended in residual liquid or in 50µL of BD Horizon Brilliant Stain Buffer (BD Biosciences) if more than one BUV fluorochrome was used. 1µL of FcR block (Miltenyi Biotec) was added followed by a Live/Dead stain as indicated in each flow cytometry panel (**Supplementary Table 2**). All other antibodies for each panel were added and cells were incubated for 10 mins at RT or 30 mins at RT if more than one BUV fluorochrome was used. Cells were acquired on a FACS Canto (BD Biosciences) or an Amnis CellStream (Luminex Corporation, Austin, TX, USA). All data analysis was performed using FlowJo software (Version 10.7.1, FlowJo LLC, Oregon, USA).

### Statistical Analysis

Differential expression of RNA sequencing reads was assessed using ANOVA, controlling for the false discovery rate using the Benjamini-Hochberg method. Statistical analysis of flow cytometry data was performed using GraphPad Prism version 9. Data were assessed for a normal distribution using the Shapiro-Wilk test and the Kolmogorov-Smirnov test. If both these tests indicated a non-normal distribution in a dataset then non-parametric statistical tests were used for the analysis. The statistical test used is indicated in each Figure legend. Within each analysis *, **, and *** represent p<0.05, p<0.01, p<0.001, respectively. A p-value of <0.05 was considered significant. Geneset and functional enrichment analysis was performed using GSEA (38) and G:Profiler (39). Tissue-residency genesets were derived from Kumar et al. (40) and Mackay et al. (41) (**Supplementary Table 3**).

## Results

### Hepatic NK Cell Subpopulations Display Transcriptional Profiles Distinct From the Corresponding Subpopulations of NK Cell in Peripheral Blood

**Figure 1:**
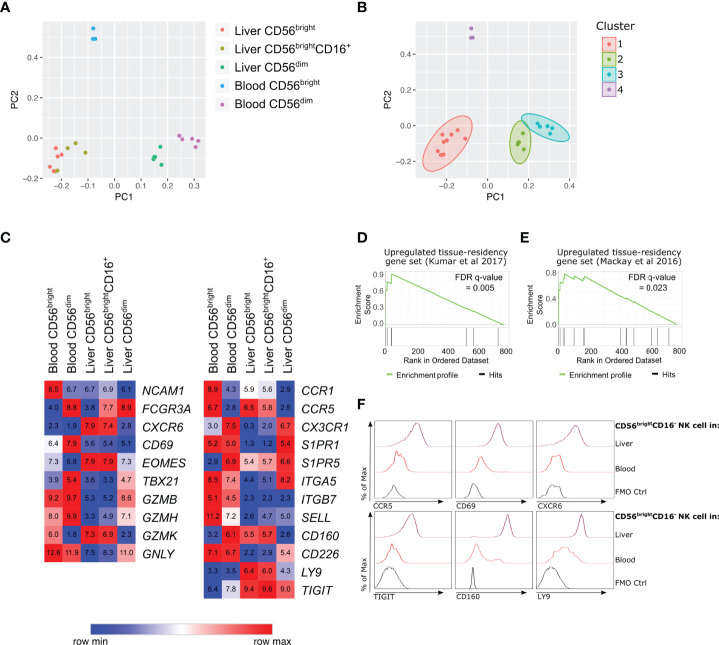
Human hepatic CD56^bright^ NK cell populations express a distinct transcriptional profile association with tissue residency. RNA-sequencing analysis of flow cytometry sorted NK cell subpopulations from healthy human liver perfusate (n = 5) and healthy human peripheral blood (n = 5). **(A)**, PCA analysis of 405 differentially expressed genes (ANOVA q-value < 0.05) and **(B)**, K-means clustering of PCA. **(C)**, Heatmap of average gene expression of selected NK cell-related genes. **(D)**, Gene-set enrichment analysis comparing 769 genes with >2 fold change between liver and blood CD56^bright^CD16^-^ NK cells, against up-regulated genes in tissue-resident lymphocytes from Kumar et al., 2017 (40). **(E)**, Gene-set enrichment analysis comparing 769 genes with >2 fold change between liver and blood CD56^bright^CD16^-^ NK cells, against up-regulated genes in tissue-resident lymphocytes from Mackay et al., 2016 (41). **(F)**, Representative histograms of protein validation of differentially regulated genes *via* flow cytometry on CD56^bright^CD16^-^ NK cells (n = 5-11).

Hepatic and peripheral blood NK cell subpopulations were sorted to >98% purity (**Supplementary Figure 1**). RNA sequencing of hepatic CD56^bright^CD16^-^, CD56^bright^CD16^+^ and CD56^dim^CD16^+^ NK cell subpopulations (n=5), as well as peripheral blood CD56^bright^CD16^-^ (n=3) and CD56^dim^CD16^+^ NK cell subpopulations (n=5), identified 405 differentially expressed genes (ANOVA q-value <0.05; **Supplementary Data File**). Principal component analysis (PCA) of these 405 genes was used to visualise the transcriptional relationships between these five NK cell subpopulations. Dimension 1 of the PCA broadly clustered the subpopulations based on CD56 expression, i.e. CD56^bright^ versus CD56^dim^ NK cells (**Figure 1A**; accounting for 51.1% of variation). A further 17.6% of the variation was explained by dimension 2, which clustered the three CD56^bright^ subpopulations on the basis of tissue of origin, i.e. blood versus liver (**Figure 1A**).

K-means clustering identified four major sample clusters (**Figure 1B**). Cluster 1 included the hepatic CD56^bright^CD16^-^ and CD56^bright^CD16^+^ samples, indicating minimal transcriptional difference between these two hepatic NK cell subpopulations (**Figure 1B**). These results support the hypothesis that hepatic CD56^bright^CD16^+^ cells arise from CD56^bright^CD16^-^, rather than representing a CD56^dim^CD16^+^ subpopulation that has upregulated CD56 expression. Clusters 2 and 3 represent hepatic CD56^dim^CD16^+^ NK cells and peripheral blood CD56^dim^CD16^+^ NK cells, respectively. These blood and liver CD56^dim^ NK cells cluster closely together, however there are sufficient transcriptional differences to separate them into distinct clusters (**Figure 1B**). Cluster 4 represents peripheral blood CD56^bright^CD16^-^ NK cells. This subpopulation is transcriptionally distinct from both CD56^dim^ NK cell populations as well as hepatic CD56^bright^ NK cells (**Figure 1B**).

### Hepatic CD56^bright^CD16^-^ NK Cells Up-Regulate Genes Associated With Tissue Residency and Possess a Unique Receptor Repertoire

Previous studies have identified hepatic CD56^bright^ NK cells as a human tissue-resident NK cell population (10–13). At a transcriptional level hepatic CD56^bright^CD16^-^ and CD56^bright^CD16^+^ NK cells both express high levels of genes encoding proteins associated with liver-resident NK cells, including *EOMES*, *CXCR6*, *CCR5* and low levels of *S1PR1* and *SELL*, which are both associated with circulating NK cell populations (**Figure 1C**). The gene expression of another tissue-residency marker, *CD69*, showed no significant difference between the five NK cell subpopulations (**Figure 1C**). This is despite significantly elevated CD69 protein expression on hepatic CD56^bright^CD16^-^ NK cells (**Figure 1F** and **Supplementary Figure 3**). The up-regulation of *CXCR6* and *CCR5* mRNA in hepatic CD56^bright^CD16^-^ NK cells was associated with high expression of the CXCR6 and CCR5 chemokine receptors on CD56^bright^CD16^-^ NK cells confirming the tissue-resident phenotype of this cell population (**Figure 1F** and **Supplementary Figure 3**).

Directly comparing hepatic CD56^bright^CD16^-^ NK cells to peripheral blood CD56^bright^CD16^-^ NK cells identified 769 genes with a geometric mean expression >10 across samples and >2 absolute fold change (**Supplementary Data File**). Amongst these 769 genes, the genes expressed higher in hepatic CD56^bright^CD16^-^ NK cells were enriched for gene signatures previously identified in tissue-resident lymphocytes (40, 41) (**Figures 1D E** and **Supplementary Table 3**), further supporting the conclusion that hepatic CD56^bright^CD16^-^ NK cells are a liver-resident NK cell population.

Functional enrichment analysis of these 769 genes identified significant enrichment of a range of immune-related processes (**Supplementary Table 4**). This included terms related to lymphocyte activation, cytokine signalling, chemokine signalling, and leukocyte cell-cell adhesion (**Supplementary Table 4**). Differentially regulated genes associated with lymphocyte activation and leukocyte cell-cell adhesion pathways included *CD160*, *CD83*, *KLRB1*, *LILRB2*, *LY9*, and *TIGIT*, which are all up-regulated on hepatic CD56^bright^CD16^-^ NK cells (**Supplementary Data**). In contrast, *CD226*, *CD44*, *CD52*, and *LGALS9* are all down-regulated on hepatic CD56^bright^CD16^-^ NK cells compared to peripheral blood CD56^bright^CD16^-^ NK cells (**Supplementary Data File**). These differences in cell surface receptors indicate that hepatic CD56^bright^CD16^-^ NK cells possess a unique repertoire of activating and inhibitory receptors and suggest that they may interact with other cell populations within the liver microenvironment, including parenchymal hepatocytes and non-parenchymal leukocyte populations.

We focussed on three of these up-regulated cell surface receptors: Ly9, TIGIT and CD160. Ly9 (also known as CD229) is a member of the SLAM receptor family expressed by lymphocyte populations and localises to the immunological synapse during cellular interactions (42). TIGIT is an inhibitory receptor, capable of binding polio-virus receptor (PVR) and nectin-2 and hepatocytes express PVR to protect themselves from NK cell-mediated killing (43). CD160 is a glycoprotein widely expressed on tissue-resident lymphocytes (**Supplementary Figure 3**). It binds both classical and nonclassical MHC class I molecules, as well as herpes virus entry mediator (HVEM), and is capable of activating NK cells (44–46). We validated the transcriptional changes in *LY9*, *TIGIT* and *CD160* at a protein level, with CD56^bright^CD16^-^ NK cells from liver perfusate samples expressing higher levels of Ly9, TIGIT and CD160 compared to peripheral blood CD56^bright^CD16^-^ NK cells (**Figure 1F** and **Supplementary Figure 3**). We also assessed the expression of these novel liver-residency associated markers on CD56^dim^NK cells, CD56^+^CD3^+^ T cells and CD56^-^CD3^+^ T cells in liver and peripheral blood samples (**Supplementary Figure 3**).

### Liver-Resident CD56^bright^ NK Cells Co-Express CD69, CXCR6, TIGIT, CD160, Ly9 and NKG2D

**Figure 2:**
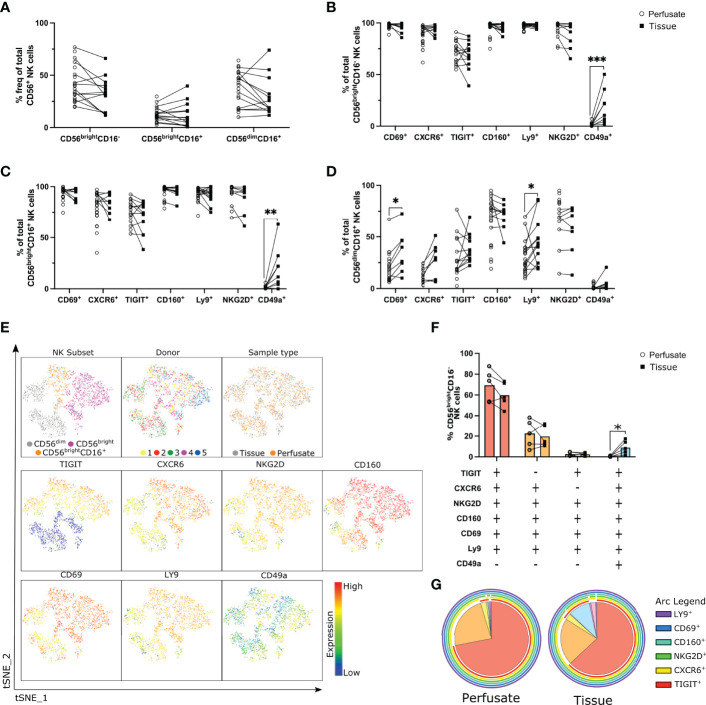
Co-expression of CD69, CXCR6, TIGIT, CD160, Ly9 and NKG2D defines a liver-resident CD56^bright^ NK cell subset. Flow cytometry analysis liver-residency associated markers on NK cell subsets in liver perfusate and tissue samples (n = 5-25). **(A)**, Frequency of NK cell subsets in liver perfusate and tissue samples (n = 5-25, matched samples indicated with a line). **(B, D)**, Percent frequency of CD69^+^, CXCR6^+^, TIGIT^+^, CD160^+^, Ly9^+^, NKG2D^+^, and CD49a^+^ CD56^bright^CD16^-^ **(B)**, CD56^bright^CD16^+^ **(C)**, and CD56^dim^CD16^-^ **(D)** NK cells in perfusate and tissue samples. **(E)**, tSNE analysis of NK cells from 5 matched liver perfusate and tissue samples visualising CD56^bright^ (CD16^-^), CD56^bright^CD16^+^, and CD56^dim^ (CD16^+^) NK cell subsets’ distinct cell surface receptor expression patterns. **(F, G)**, SPICE analysis visualising co-expression of cell surface receptors on CD56^bright^CD16^-^ NK cells in matched liver perfusate and tissue samples represented as a scatter plot **(F)** and a pie chart **(G)**. Data analysed using an unpaired Mann-Whitney test **(B–D)** or a paired t test **(F)**. * p< 0.05, **p < 0.01, ***p < 0.001.

To confirm the high expression of TIGIT, CD160, Ly9 on liver-resident NK cell populations we next investigated hepatic NK cell subsets in liver perfusate and tissue samples. There was no difference in frequencies of NK cell subsets in liver perfusate and tissue samples (**Figure 2A**). This aligns with previous studies that have shown that the lymphocyte subpopulations present in liver perfusate closely resembles those directly isolated from liver tissue (10). We assessed the expression of liver-residency associated markers (CD69, CXCR6), the novel receptors identified from our transcriptional analyse (TIGIT, CD160, Ly9), as well as the integrin CD49a and the activating receptor NKG2D, on hepatic NK cell subsets (n=5-25). CD56^bright^CD16^-^ and CD56^bright^CD16^+^ NK cell subsets from both liver perfusate and tissue samples expressed high levels of CD69, CXCR6, CD160, Ly9, and NKG2D (**Figures 2B, C**). TIGIT was expressed by a slightly lower percentage of CD56^bright^CD16^-^ (72.25% ± 10.75 in perfusate and 68.38% ± 13.08 in tissue; mean ± SD; **Figure 2B**) and CD56^bright^CD16^+^ cells (75.27% ± 11.72 in perfusate and 70.66% ± 15.43 in tissue; **Figure 2C**), but there was no significant difference between liver perfusate or liver tissue.

The only cell surface marker we identified that showed a difference between liver perfusate and liver tissue was CD49a. CD49a was not expressed on CD56^bright^CD16^-^ or CD56^bright^CD16^+^ NK cells isolated from liver perfusate (**Figures 2B, C**). Populations of CD49a^+^CD56^bright^CD16^-^ and CD49a^+^CD56^bright^CD16^+^ NK cells were present in liver tissue and the frequency of these populations was very variable between samples (1.84-50.20% and 0-63.10%; respectively; min-max range; **Figures 2B, C**). CD49a^+^ NK cells have been described in both the mouse and the human liver (47, 48), although this population is highly variable between donors in human liver. Our data indicates this CD49a^+^ population of hepatic NK cells is only detectable in tissue and not liver perfusate.

Interestingly, significantly higher frequencies of CD69^+^ and Ly9^+^ CD56^dim^CD16^+^ NK cells were present in liver tissue samples versus perfusate (p=0.0325 and p=0.0149 respectively; **Figure 2D**). This indicates that although CD56^dim^ NK cells are not considered a liver-resident population, subsets of hepatic CD56^dim^ NK cells may up-regulate markers associated with tissue-residency in the liver.

These subtle differences in receptor frequencies between liver tissue and liver perfusate open the possibility that a specific subpopulation of NK cells may be present in liver tissue and absent in perfusate. To explore this possibility a t-distributed stochastic neighbour embedding (tSNE) analysis was carried out on CD56^+^CD3^-^ NK cells from 5 matched liver perfusate and tissue samples (**Figure 2E**). Using CD56, CD16, CXCR6, CD160, TIGIT, NKG2D, LY9, CD69, and CD49a NK cell subsets clustered separately into CD56^bright^ (purple), CD56^bright^CD16^+^ (orange), and CD56^dim^ (grey) NK cell clusters (**Figure 2E**). There was no clustering observed between donors or sample type indicating minimal differences in receptor expression on NK cell subpopulations obtained from different donors (**Figure 2E**). This also indicates that the NK cell subpopulations detectable in liver perfusate are broadly representative of digested tissue. Both the CD56^bright^ and CD56^bright^CD16^+^ NK cell clusters express higher levels of CXCR6, CD160, TIGIT, NKG2D, Ly9 and CD69 in comparison to the CD56^dim^ NK cell subset, and CD49a expression is low across all subsets (**Figure 2E**).

To explore receptor co-expression we utilised Simplified Presentation of Incredibly Complex Evaluations (SPICE) (49) plots to visualise the co-expression on CD56^bright^CD16^-^ NK cell subsets from both perfusate and tissue samples (**Figures 2F, G**). The majority of CD56^bright^CD16^-^ NK cells in both perfusate and tissue samples are TIGIT^+^CXCR6^+^NKG2D^+^CD160^+^CD69^+^Ly9^+^CD49a^-^ (red segment; 70% ± 15.32% and 58.28% ± 12.88% respectively) (**Figures 2F, G**). The second most frequent subset differs only in regard to TIGIT expression (orange segment; TIGIT^-^CXCR6^+^NKG2D^+^CD160^+^CD69^+^Ly9^+^CD49a^-^; 20.68 ± 15.05% and 21.43 ± 11.98% respectively) (**Figures 2F, G**). These data indicate that the majority of liver-resident CD56^bright^CD16^-^ NK cells co-express the cell surface receptors CD160 and Ly9, in addition to CXCR6, CD69 and NKG2D, and this population is present in both liver perfusate and tissue. A small population of CD49a^+^CD56^bright^CD16^-^ NK cells, which also co-express markers of tissue-residency, are only detectable in digested liver tissue (**Figures 2F, G**).

### Hepatic NK Cells But Not Peripheral Blood NK Cells Kill Activated Allogenic Peripheral Blood CD8^+^ T Cells

**Figure 3:**
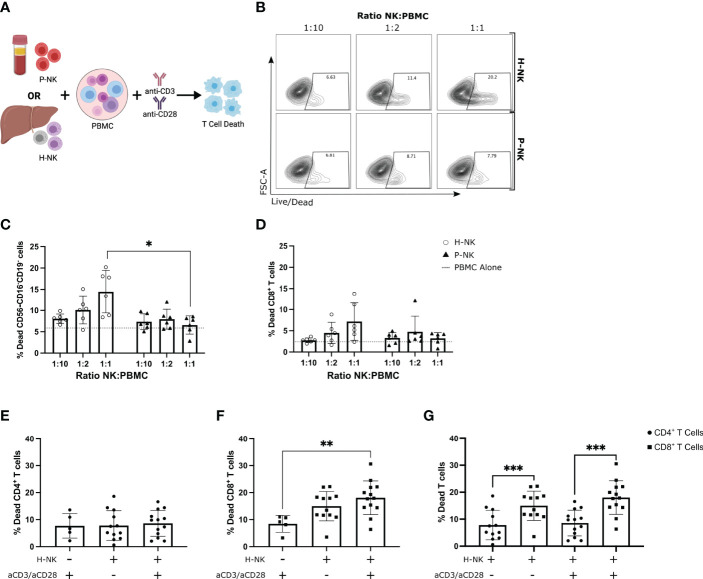
Hepatic NK cells but not peripheral blood NK cells dose-dependently kill activated allogenic peripheral blood CD8^+^ T cells. PBMCs cultured alone or with magnetically sorted hepatic NK cells (H-NK) or peripheral blood NK cells (P-NK) cells for 24 hr with or without anti-CD3/28 activation stimulation (n = 6-13). **(A)**, Schematic of experimental design. **(B)**, Representative flow plots of % dead CD56^-^CD16^-^CD19^-^ cells at each indicated ratio for both H-NK and P-NK cocultures. **(C, D)**, Percent dead CD56^-^CD16^-^CD19^-^ cells **(C)** and CD56^-^CD16^-^CD19^-^CD8^+^ cells **(D)** after 24 hr co-culture with H-NK (clear circle) or P-NK (triangle). Grey dashed line represents PBMCs cultured alone. **(E–G)**, Percent dead CD4^+^ T cells **(E)** and CD8^+^ T cells **(F)** post-PBMC culture alone or with H-NK cells and/or anti-CD3/28 activation (n = 12-13) **(G)**, Data from **(E, F)** directly comparing CD4^+^ T cell and CD8^+^ T cell death post-coculture with or without anti-CD3/CD28 activation. Data analysed using a 2-Way ANOVA **(C, D)**, a one-way ANOVA **(E, F)** or a paired t test **(G)**. *p<0.05, ** p<0.01, and ***p<0.001.

The co-expression of receptors involved in lymphocyte activation and leukocyte cell-cell adhesion pathways highlights that liver-resident NK cell populations may interact in important way with other cell types in the liver microenvironment. Previous studies have identified a role of NK cells in regulating T cell activation in liver disease (19, 20) and based on the receptor co-expression we hypothesised that this functional role may be of relevance to immune tolerance in the liver during transplantation. Activated PBMCs were cultured with or without total hepatic NK cells or total peripheral blood NK cells at increasing ratios for 24 hr and T cell death was analysed by flow cytometry (n=6; **Figure 3A**). As T cells downregulate their CD3 expression during early stages of activation, a gating strategy was employed to exclude any B and NK cells from our analysis of T cell death (**Supplementary Figure 4A**). After excluding B and NK cells, CD56^-^CD16^-^CD19^-^ lymphocyte cell death increased in both the hepatic and peripheral blood NK cell co-cultures versus T cells alone (**Figure 3C**). Cell death was significantly higher in hepatic NK cell cocultures in comparison to peripheral blood NK cells cocultured with PBMCs at a 1:1 ratio (p=0.0306; **Figure 3C**). Furthermore, only the hepatic NK cell co-cultures resulted in a significant dose-dependent increase in cell death with increasing numbers of NK cells (2-way ANOVA, ratio and NK origin interaction p=0.0032; **Figure 3B, C**). This significant dose-dependent hepatic NK cell killing effect was also evident when analysing CD8+ T cells specifically (2-way ANOVA, ratio and NK origin interaction p=0.043; **Figure 3D**). This indicates that hepatic NK cells have an enhanced ability to kill activated allogenic T cells in comparison to peripheral blood NK cells.

To assess if the hepatic NK cell killing effect is specific to a subset of T cells, hepatic NK cells were co-cultured with PBMCs with and without anti-CD3/CD28 stimulation and both CD8^+^ T cell and CD4^+^ T cell death was assessed (n=12-13; **Figures 3E-G**). A similar gating strategy was employed with the addition of an anti-CD4 conjugated-fluorochrome (**Supplementary Figure 4B**). There was no difference in CD4^+^ T cell death in hepatic NK cell and PBMC co-cultures regardless of anti-CD3/CD28 activation (**Figure 3E**). CD8^+^ T cell death was significantly higher in hepatic NK cocultures that were activated with anti-CD3/CD28 in comparison to activated PBMCs cultured alone (p=0.0049; **Figure 3F**). When directly compared, CD8^+^ T cell death was higher than CD4^+^ T cell after coculture with hepatic NK cells in both non-activated and activated groups (p=0.0009 and p<0.0001 respectively; **Figure 3G**). These results indicate hepatic NK cells preferentially kill allogenic CD8^+^ T cells over CD4^+^ T cells.

### CD160 Engagement Enhances Killing of Allogenic T Cells by Hepatic NK Cells

As liver-resident NK cells highly express CD160, Ly9, TIGIT and NKG2D we hypothesised that they may have a role in regulating hepatic CD56^bright^ NK cell activation in response to activated allogenic CD8^+^ T cells. Specific antagonist monoclonal antibodies targeting human Ly9 or CD160 are not available, however an agonist monoclonal antibody targeting CD160 has been described (50, 51). Hepatic NK cells were cocultured with PBMCs for 24 hr in the presence of either an agonistic mAb against CD160, an antagonistic mAb against TIGIT or NKG2D, a recombinant Ly9 protein or an IgG1 or IgG2A isotype control (**Supplementary Figure 5**; n=11-13). The addition of the recombinant Ly9 protein or mAbs against NKG2D, TIGIT, IgG1 or IgG2A isotype control had no effect on either CD8^+^ or CD4^+^ T cell death (**Supplemental Figures 5B, C**). Addition of the agonistic anti-CD160 mAb significantly increased CD8^+^ T cell death (p=0.0343). A trend towards increased CD4^+^ T cell death was observed but this failed to reach statistical significance (p=0.4539; **Supplementary Figures 5B, C**). This increase is T cell death was only observed in the presence of hepatic NK cells. Addition of the agonistic anti-CD160 mAb to T cells alone did not result in an increase in CD4^+^ or CD8^+^ T cell death (**Supplementary Figures 5D, E**).

**Figure 4:**
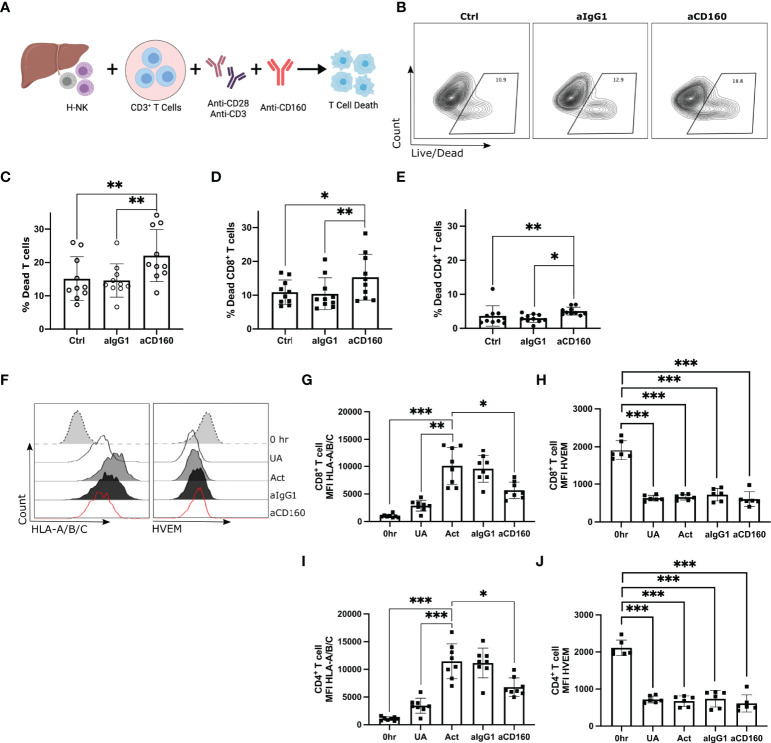
CD160 engagement on hepatic NK cells enhances killing of allogenic CD8^+^ T cells. Activated CD3^+^ T cells co-cultured with H-NK cells with or without a mAb against CD160 for 24 hr (n = 13). **(A),** Schematic of experimental design. **(B)**, Representative flow plots of the percent dead total T cells in each treatment group. ‘Ctrl’ indicates cocultures of CD3^+^ T cells with H-NK cells without the addition of any antibody. ‘aIgG1’ indicates cocultures of CD3^+^ T cells with H-NK cells with the addition of an IgG1 antibody. ‘aCD160’ indicates cocultures of CD3^+^ T cells with H-NK cells with the addition of an anti-CD160 mAb. **(C–E)**, Percent dead total T cells **(C)**, CD8^+^ T cells **(D)** and CD4^+^ T cells **(E)** following 24 hr co-culture of CD3^+^ T cells with hepatic NK cells. **(F),** Representative flow plots of HLA-A/B/C and HVEM MFI of CD8+ T cells at 0 hr and for each treatment at 24 hr. **(G, H)**, MFI values for HLA-A/B/C **(G)** and HVEM **(H)** on CD8^+^ T cells and CD4^+^ T cells at 0 hr and for each treatment group at 24 hr. Data analysed using a Freidman test with Dunn’s multiple comparison **(C–E)** or a repeated measures one-way ANOVA **(G–J)**. *p < 0.05, ** p < 0.01, and ***p < 0.001.

To focus further on this CD160-mediated mechanism of enhanced CD8^+^ T cell death, CD3^+^ T cells were magnetically sorted from PBMCs prior to co-culture with hepatic NK cells and the mAb against CD160 (**Figure 4A**; n=12). After 24 hr T cell death was assessed by flow cytometry (**Supplementary Figure 4B**). Addition of the anti-CD160 mAb significantly increased the total T cell death (median 12.45% versus 19.10%, p=0.001; **Figures 4B,C**), CD8^+^ T cell death (median 10.33% versus 13.95%, p=0.0219; **Figure 4D**) and CD4^+^ T cell death (median 2.57% versus 4.59%, p=0.0052; **Figure 4E**).

We next investigated the potential receptor-ligand interaction involved in this CD160-mediated enhancement of CD8^+^ T cell death. We measured the expression of known CD160 ligands, HVEM and HLA class-I molecules (44–46), on CD8^+^ T cells and CD4^+^ T cells at 0 hr and at 24 hr post-treatment (n=6-8; **Figures 4F-H**). Coculture of hepatic NK cells and CD3^+^ T cells without activation resulted in a small increase in CD8^+^ T cell or CD4^+^ T cell HLA class-I expression. Activation with anti-CD3/CD28 significantly increased T cells’ HLA class-I expression (**Figures 4F, G, I**). The addition of the anti-CD160 mAb, which results in enhanced T cell death, significantly decreased both CD4^+^ and CD8^+^ T cell expression of HLA class-I (**Figures 4F, G, I**). Freshly isolated CD8^+^ T cells and CD4^+^ T cells expressed high levels of HVEM but downregulated HVEM expression post-coculture with hepatic NK cells regardless of anti-CD3/CD28 stimulation (**Figures 4F, H, J**).

### Hepatic CD56^bright^ NK Cells Are Responsible for the CD160-Mediated Increase in T Cell Death

**Figure 5:**
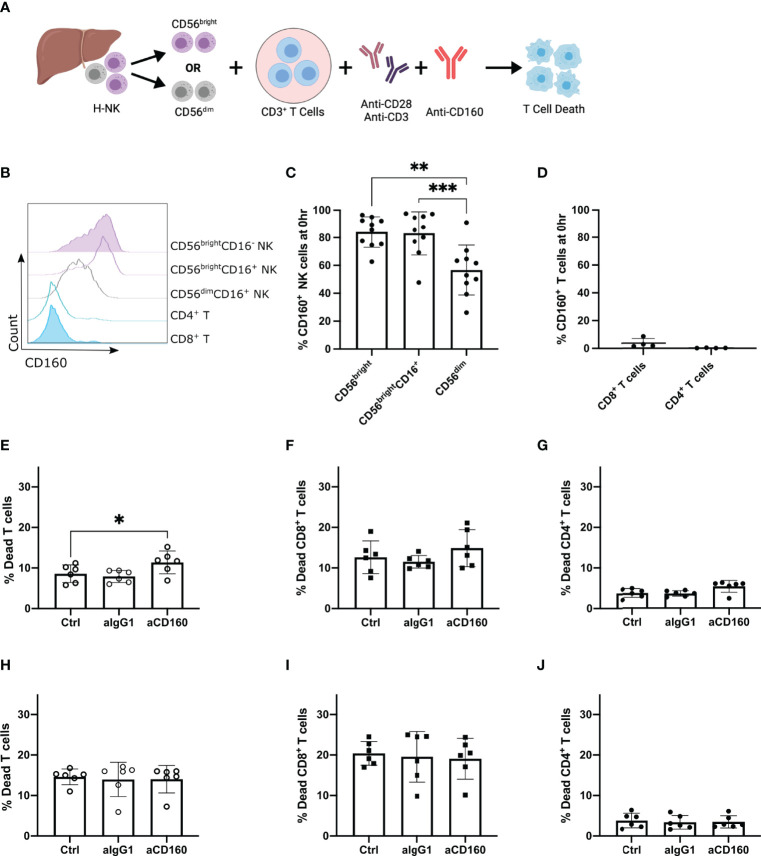
Agonistic anti-CD160 mAb acts on hepatic CD56^bright^ NK cells to enhance their killing of allogenic T cells. Activated CD3^+^ T cells co-cultured with H-NK cells or FACS-sorted hepatic CD56^bright^CD16^+/-^ or CD56^dim^CD16^+^ NK cells with or without a mAb against CD160 for 24 hr (n = 6-10). **(A)**, Schematic of experimental design **(B),** Representative flow plot of CD160 expression on NK and T cell populations at 0 hr prior to coculture. ‘Ctrl’ indicates cocultures of CD3^+^ T cells with the specified H-NK cell subset without the addition of any antibody. ‘aIgG1’ indicates cocultures of CD3^+^ T cells with the specified H-NK cell subset with the addition of an IgG1 antibody. ‘aCD160’ indicates cocultures of CD3^+^ T cells with the specified H-NK cell subset with the addition of an anti-CD160 mAb. **(C, D),** Percent CD160 expression on NK cell **(C)** and T cell **(D)** subsets at 0 hr. **(E–G),** Percent dead total T cells **(E)**, CD8^+^ T cells **(F)** and CD4^+^ T cells **(G)** following 24 hr co-culture of CD3^+^ T cells and CD56^bright^CD16^+/^- hepatic NK cells. **(H–J)**, Percent dead CD56^-^CD19^-^ cells **(H)**, CD8^+^ T cells **(I)** and CD4^+^ T cells **(J)** following 24 hr co-culture of CD3^+^ T cells with hepatic CD56^dim^CD16^+^ NK cells. Data analysed using a repeated measures One Way ANOVA test **(C–J)**. * p< 0.05, **p < 0.01, and ***p < 0.001.

As hepatic NK cells consist of variable frequencies of both CD56^bright^CD16^-^, CD56^bright^CD16^+^ and CD56^dim^CD16^+^ NK cell populations, we next FACS-sorted hepatic NK cells into CD56^dim^ (CD56^dim^CD16^+^) and CD56^bright^ (CD56^bright^CD16^+/-^) NK cell subpopulations to confirm that the effect of CD160 engagement was specific to either the CD56^bright^ or CD56^dim^ hepatic NK cell subset (n=6, **Figure 5A**). The expression of CD160 was measured on hepatic NK and blood T cells prior to coculture at 0 hr as although CD160 is highly expressed by liver-resident CD56^bright^ NK cells it has also been described to be expressed by CD56^dim^ NK cells and a minor subset of CD8^+^ T cells in the blood (52–54). In hepatic samples CD56^bright^CD16^-^ and CD56^bright^CD16^+^ NK cells expressed higher levels of CD160 in comparison to CD56^dim^ NK cells (p=0.003 and p=0.0003 respectively; **Figures 5B, C**). Conversely, CD4^+^ T cells did not express CD160 and frequencies of CD160^+^CD8^+^ T cells were low (mean ± SD 3.72 ± 3.33%; **Figures 5B, D**). We therefore hypothesised that the agonistic mAb against CD160 is acting directly on the hepatic CD160^+^CD56^bright^CD16^+/-^ NK cells in our coculture system.

Addition of the CD160 mAb to the CD56^bright^ co-culture resulted in a significant increase in total T cell death (p=0.0382; **Figure 5E**), and a trend towards an increase in both CD4^+^ and CD8^+^ T cell death (p=0.0526 and p=0.1778 respectively; **Figures 5F, G**). In contrast, addition of the CD160 mAb to CD56^dim^ co-cultures had no effect on CD3^+^ T cell death (**Figures 5H–J**). These results indicate that hepatic CD56^bright^ NK cell-mediated cytotoxicity is enhanced by CD160 engagement but CD56^dim^ NK cell-mediated cytotoxicity is not. Interestingly overall CD3^+^ T cell death was slightly higher in hepatic CD56^dim^ (14.6 ± 1.94%; **Figure 5H**) co-cultures in comparison to hepatic CD56^bright^ (8.598 ± 2.18%; **Figure 5E**). This result highlights that both hepatic CD56^bright^ and CD56^dim^ NK cells contribute to the overall ability of hepatic NK cells to kill allogenic CD8^+^ T cells, however only CD56^bright^ NK cells are activated *via* CD160 ligation.

## Discussion

In this study, we show that hepatic CD56^bright^CD16^+/-^ NK cells are distinct from their circulating CD56^bright^ counterparts and hepatic CD56^dim^ NK cells with a transcriptional profile that is indicative of tissue residency. The majority of CD56^bright^ NK cells in both human liver perfusate and tissue are liver-resident NK cells that co-express CD69, CXCR6, NKG2D, CD160, and Ly9, lack expression of the integrin CD49a, and have variable expression of TIGIT. We show for the first time that hepatic NK cell populations kill activated allogenic CD8^+^ T cells and that this killing can be enhanced by the engagement of CD160 on CD56^bright^ liver-resident NK cells.

The ability of hepatic NK cell populations to directly target allogenic CD8^+^ T cells implicates liver-resident NK cells as important regulators of immune tolerance in the human liver. This is of particular importance in liver-transplantation where CD8^+^ T cells are involved in mediating rejection episodes (55, 56). Previous studies have suggested that NK cells may act as biomarkers of operational tolerance in liver transplant patients, defined as stable graft function in liver transplant recipient in the absence of immunosuppressive drugs (57, 58). Transcriptional profiling of operationally tolerant liver transplant patients identified an NK-related gene signature associated with tolerance (59, 60) and an expansion of NK cells in peripheral blood has been associated with allograft acceptance (27, 61). The ability of hepatic NK cells to kill allogenic activated CD8^+^ T cells provides biological rationale for these findings and indicates that quantification of liver-resident NK cell frequency and function may help to predict the development of operational tolerance in liver transplant recipients. Future research should address whether similar results are observed in autologous models.

The activating receptor CD160 is highly expressed by liver-resident CD56^bright^ NK cells. Studies have demonstrated that triggering CD160 on NK cells drives NK cell activation (44–46), which contrasts with the inhibitory effect of CD160 signalling on T cells where it acts to block proliferation and cytokine production (53, 62–64). We validated the functional significance of the activating receptor CD160 on liver-resident CD56^bright^ NK cells using an agonistic anti-CD160 mAb in a co-culture model. We have demonstrated that CD160 engagement enhances liver-resident CD56^bright^ NK cells’ killing of both CD4^+^ and CD8^+^ T cells but did not enhance cytotoxicity by the hepatic CD56^dim^ NK cells, which express lower levels of the CD160 receptor. Upon activation both CD4^+^ and CD8^+^ T cells upregulate class I HLA molecules, which are recognised by CD160, indicating additional unknown receptor-ligand interactions are responsible for the preferential killing of allogenic CD8^+^ T cells. Our findings are limited by a lack of antagonistic monoclonal antibodies targeting human CD160. Further studies are required to confirm the mechanism of preferential CD8+ T cell recognition/killing, and the potential role that CD160 plays in recognition of T cells by liver-resident CD56^bright^ NK cell.

The expression of the inhibitory receptor TIGIT was variable among liver-resident CD56^bright^ NK cells which may be a mechanism of regulating hepatic tolerance. Engagement of TIGIT with its ligands, PVR and nectin-2, results in the inhibition of NK cell cytotoxicity and IFN-γ production (65–67). During liver regeneration, hepatocytes have been shown to increase PVR to protect themselves from NK cell-mediated killing (43). The local availability of TIGIT ligands may therefore directly influence NK cell function. Ly9 (also known as CD229) is a member of the SLAM receptor family and was also highly expressed by liver-resident CD56^bright^ NK cells in our study. The Ly9 receptor acts as a self-ligand and when bound to another Ly9 molecule a signalling cascade is initiated which involves the recruitment of adaptor proteins SAP and EAT-2 (68, 69). The functional role of Ly9 on NK cells is unknown; however, a Ly9-engineered CAR T cell therapy has shown beneficial anti-tumour effect in murine models by targeting CD229^hi^ myeloma cells (70) which suggests Ly9 acts as an activating receptor. We show that Ly9 is highly expressed by other hepatic immune cell populations and future research is needed to investigate its role in mediating cellular interactions in the human liver.

While liver-resident CD56^bright^ NK cells represent the largest population of hepatic NK cells, it is evident that this subset is just one component of an extensive repertoire of hepatic NK cell populations. In this study we identified a population of CD56^bright^CD16^+^ NK cells in the human liver. This population is generally present at low frequencies in the liver but is absent from peripheral blood. We demonstrate that this CD56^bright^CD16^+^ NK cell population has a similar transcriptional profile to hepatic CD56^bright^CD16^-^ NK cells. This suggests that the CD56^bright^CD16^+^ NK cells may arise from CD56^bright^ NK cells, by up-regulating CD16 expression under specific conditions in the liver microenvironment. CD16 expression is inducible on CD56^bright^CD16- NK cells and is driven by IL-15 signalling and the presence of accessory cells (71). Interestingly, IL-15 signalling has been identified as important in inducing autophagy, which is required for tissue-residence programming and mitochondrial fitness in human liver-resident memory CD8^+^ T cells (72) and we have previously demonstrated significant amounts of IL-15 protein in human liver (73).

A subset of CD49a^+^CD56^bright^ NK cells was also identified in this study and has been described previously (47, 48). CD49a is the α1 subunit of the integrin α1β1 that interacts with collagen IV and laminin, which are major components of the extracellular matrix that are abundant in the liver (74). Murine liver-resident NK cells are CD49a^+^ and display strong cytokine responses to stimulation (75,76). Human CD49a^+^ NK cells display stronger cytokine responses but a weaker cytotoxic response than CD49a^-^ NK cells (47). Marquardt et al. identified CD49a^+^CD56^bright^ NK cells in only 12 out of 29 livers examined and overall these cells represented an average of 2.3% of total hepatic CD56^+^ NK cells (47). In our study CD49a^+^CD56^bright^ NK cells were present in all 8 healthy tissue samples yet their frequency was highly variable between donors (1.84-50.20% min-max range). Stary et al. identified CD49a^+^CD56^bright^ NK cells in 27 resected tissue samples where they represented an average of 30% of total hepatic NK cells (circa 5-60% min-max range) (48). Interestingly, both studies measured tissue-derived CD49a^+^CD56^bright^ NK cells’ T-bet and Eomes expression. Marquardt et al. described these cells as bona fide T-bet^+^Eomes^-^, whereas Stary et al. observed a Eomes^hi^T-bet^lo^ phenotype. We did not measure T-bet and Eomes expression in our CD49a^+^ NK cell subsets, however these studies suggest that multiple CD49a^+^CD16^-^ NK cell populations may exist in the human liver. In our study, CD49a^+^CD56^bright^ NK cells also co-expressed the functional receptors NKG2D, LY9, CD160 and TIGIT, and further studies of their functional roles in human liver are warranted.

Overall, our study supports the hypothesis that liver-resident CD56^bright^ NK cells are immunoregulatory cells that play an important role in the regulation of allogenic CD8^+^ T cell populations in the liver. Understanding the tolerogenic mechanisms of the human liver is important for the development of novel strategies for enhancing allograft acceptance and reducing immunosuppression-associated comorbidities and mortality rates amongst transplant recipients. We show here that liver-resident CD56^bright^ NK cells are equipped with the functional receptors TIGIT, CD160 and Ly9 and have the ability to kill activated allogenic CD8^+^ T cells. We propose that liver-resident NK cell cytotoxicity of allogenic CD8^+^ T cells contributes to the liver’s tolerogenic immune microenvironment post-transplantation, perhaps explaining why these patients need far less immunosuppression than recipients of other solid organs.

## Data Availability Statement

The datasets presented in this study can be found in online repositories. The names of the repository/repositories and accession number(s) can be found in the article/**Supplementary Material**.

## Ethics Statement

The studies involving human participants were reviewed and approved by St. Vincent’s University Hospital Ethics Committee. The patients/participants provided their written informed consent to participate in this study.

## Author Contributions

GJ, MR, and CO’F designed and managed the project. GJ, RS, and MR performed the experimental research. GJ and MR performed statistical analysis and data interpretation. DH and TG recruited participants, acquired biological samples and assisted in cohort profiling. GJ, CH, LL, MR, and CO’F contributed to interpretation of data and writing of the manuscript. All authors contributed to the article and approved the submitted version.

## Funding

This research was funded by the Health Research Board (grant number EIA-2017-013).

## Conflict of Interest

The authors declare that the research was conducted in the absence of any commercial or financial relationships that could be construed as a potential conflict of interest.

## Publisher’s Note

All claims expressed in this article are solely those of the authors and do not necessarily represent those of their affiliated organizations, or those of the publisher, the editors and the reviewers. Any product that may be evaluated in this article, or claim that may be made by its manufacturer, is not guaranteed or endorsed by the publisher.
