# Supplementary material for: Human Hepatic CD56bright NK Cells Display a Tissue-Resident Transcriptional Profile and Enhanced Ability to Kill Allogenic CD8+ T Cells
Source: Front Immunol. 2022 Jul 5;13:921212. doi: 10.3389/fimmu.2022.921212 (PMC9295839; doi:10.3389/fimmu.2022.921212)
Supplement: Supplementary file 1 [file DataSheet_1.pdf]

Supplementary Material

1 Supplementary Data

Supplementary Data is located in the 'Supplementary_Data.xlsx' file.

2 Supplementary Tables

Supplementary Table 1: Kits and reagents

	Item	Code	Brand	Supplier
**Media/Buffers**	RPMI 1640 Medium, GlutaMAX™ Supplement	61870-010	Gibco™	Thermo Fisher Scientific
	Penicillin-Streptomycin (10,000 U/mL)	15140122	Gibco™	Thermo Fisher Scientific
	Fetal Bovine Serum	F9665	Sigma-Aldrich	Merck
	Ficoll-Paque™ PLUS Media	11778538	Cytiva	GE Healthcare
	Hank's 1X Balanced Salt Solution	11546291	HyClone™	GE Healthcare
	Bovine Serum Albumin (BSA)	12330023	HyClone™	GE Healthcare
	Molecular grade water	10787944	HyClone™	GE Healthcare
	FcR Blocking reagent	130-059-901	Miltenyi Biotech	Miltenyi Biotech
	BD Horizon Brilliant Stain Buffer	563-794	BD Biosciences	BD Biosciences
**Chemicals**	Dimethyl Sulfoxide	10103483	Fisher Bioreagents	Thermo Fisher Scientific
	EDTA	15575020	Invitrogen	Thermo Fisher Scientific
	DNAse I	9003-98-9	Sigma Aldrich	Merck
	Tumor digestion kit	130-095-929	Miltenyi Biotech	Miltenyi Biotech
	Potassium hydrogen carbonate,	15149724	ACROS Organics	Thermo Fisher Scientific
	Ammonium chloride,	10296920	ACROS Organics™	Thermo Fisher Scientific
**Kits**	EasySep™ Human NK Cell Isolation Kit	17955	STEMCELL	STEMCELL
	RosetteSep™ Human NK Cell Isolation	15025	STEMCELL	STEMCELL

	EasySep™ Human CD3$^+$ T Cell Isolation Kit	17851	STEMCELL	STEMCELL
**Functional Antibodies**	αCD3	40-0037-U500	Tonbo Biosciences	Tonbo Biosciences
	αCD28	40-0289-U500	Tonbo Biosciences	Tonbo Biosciences
	αCD160 clone CL1-R2	K0122-1	MBL	Caltag
	Purified Mouse IgG1, κ Isotype Ctrl Antibody	400102	Biolegend	MSC
**Cytokines**	IL-15	130-095-762	Miltenyi	Miltenyi

Supplementary Table 2: Flow cytometry antibodies and viability stains

Target	Indicated Panel	Clone	Fluorophore	Code	Supplier
**CD127**	RNA-Seq sort	REA614	APC-Vio770	130-109-438	Miltenyi Biotech
**CD3**	RNA-Seq sort	UCHT1	BV421	BD 562426	BD
**CD45**	RNA-Seq sort	HI30	BV510		BD
**CD56**	RNA-Seq sort	NCAM16.2	BV711	BD 563169	BD
**CD16**	RNA-Seq sort	3G8	PE-Cy7	BD 557744	BD
**Dead cells**	RNA-Seq sort	-	Propidium iodide	P1304MP	Thermo Fisher Scientific
**Dead cells**	NK and T cell cocultures	-	Zombie Yellow	423104	BioLegend
**Dead cells**	Phenotyping	-	Zombie NearIR	423106	BioLegend
**CD56**	NK and T cell cocultures /Phenotyping	AF12-7H3	VioBright FITC	130-113-871	Miltenyi
**CD16**	NK and T cell cocultures /Phenotyping	3G8	Pe-Cy7	302016	BioLegend
**CD19**	NK and T cell cocultures	HIB19	PerCpcy5.5	TO0265-0199-T100	Tonbo
**CD8**	NK and T cell cocultures	SK1	APC-Cy7	557834	BD
**CD4**	NK and T cell cocultures	RPA-T4	PE	60-0049-T100	Tonbo BioSciences
**CXCR6**	Phenotyping	K041E5	PerCpCy5.5	356010	BioLegend
**CD160**	Phenotyping	BY55	AF647	341204	BioLegend
**CD3**	Phenotyping	HIT3a	PacBlue	300330	BioLegend
**CD69**	Phenotyping	FN50	BV510	310936	BioLegend
**LY9**	Phenotyping	HLy9.1.25	BV605	745243	BD
**NKG2D**	Phenotyping	1D11	BV711	563688	BD
**TIGIT**	Phenotyping	A15153G	PE	372704	BioLegend
**CD49a**	Phenotyping	REA1106	PE-Vio615	130 119 308	Miltenyi
**CD8**	Phenotyping	RPA-T8	BUV395	563795	BD
**CD127**	Phenotyping	A019D5	BV570	351307	BioLegend

Supplementary Table 3: Tissue residency gene sets

Publication	Details	Genes
Kumar *et al.* 2017 (doi: 10.1016/j.celrep. 2017.08.078)	Up regulated in human tissue-resident CD8+CD69+ populations	ARHGAP18, CCR1, CRTAM, TMIGD2, IL17RE, KCNK5, IL23R, TRPM2, ADAM12, IRF4, RGS1, KCNQ3, COL5A1, FSD1, CSF1, DAB2IP, IL10, MCAM, CPNE7, ATP8B4, CXCR6, ITGAE, SPRY1, SCUBE1, ITGA1, GSG2, IL17A, CXCL13, CA10
Kumar *et al.* 2017 (doi: 10.1016/j.celrep. 2017.08.078)	Down regulated in human tissue-resident CD8+CD69+ populations	LRFN2, DGKK, FGFBP2, SLC1A7, TXNDC3, CX3CR1, KIR3DX1, KRT73, KIF19, PRSS23, SH3RF2, RAP1GAP2, SGCD, EPHX4, KRT72, ASCL2, PTGDS, PODN, CXCR2, EFHC2, LAIR2, SOX13, C1orf21, PALLD, NUAK1, COL6A2, TKTL1, PCDH1, ZNF711, SELL, S1PR1, DNAI2, FLJ13197, FAM19A1, LILRB1, GPR56, SELP, DCHS1, OSBPL5, FCGR3A, MSX2P1, SLCO4C1, PLEKHG3, D4S234E, PDZD4, SPTB, EBF4, PATL2, LOXL4, ITGAM, NPDC1, TTYH2, ME3, TTC16, SBK1, GPA33, VCL, ZNF365, KLF3, SSBP3, FZD4, KLF2, FAM65B, KIR2DS4, TFCP2L1, MTSS1, TMCC3, CFH, AGPAT4, SVIL, HPCAL4, TSPAN18, STK38, SSX2IP, NHSL2, ADAMTS10, GNLY, PELI2
Mackay *et al.* 2016 (doi: 10.1126/ science.aad2035)	Up regulated in murine tissue-resident memory T cells	ABI3, ARRDC3, ATF3, B4GALNT4, BTG2, CD244, CD69, CDH1, CISH, CSRNP1, CTNNA1, CXCR6, DDX3X ,DGAT1, DHCR24 ,DUSP1, DUSP5, DUSP6, EGR1, EHD1, EYA2, FOS, FOSB, FOSL2, FRMD4B, GADD45B, GLRX, GPR171, GPR34, GPR55, HILPDA, HMGCS1, HPGDS, HSPA5, HSPD1, IFNG, INPP4B, INSIG1, IRF4, ISG20, ITGAE, JUN, JUNB, KLF6, LAD1, LDLRAD4, LITAF, LY6G5B, MAPKAPK3, NEDD4, NEURL3, NFKBID, NR4A1, NR4A2, ODC1, OSGIN1, P2RY10, P4HB, PER1, PLK3, PNRC1, PPP1R15A, PPP1R16B, PYGL, QPCT, RGS2, RHOB, RNF149, SIK1, SKIL, SMIM3, SPSB1, STARD4, TNFAIP3, TRAF4, VDAC1, XCL1, ZFP36, AMICA1, GPR56, GSG2, SC4MOL
Mackay *et al.* 2016 (doi: 10.1126/ science.aad2035)	Down regulated in murine tissue-resident memory T cells	AAED1, ABHD8, ABTB2, ACP5, ARHGAP26, ARHGEF18, ASRGL1, ATP10D, ATP1B3, AVEN, B3GAT3, BCL9L, CCL5, CD84, CDC25B, CXCR4, DOCK2, EHD3, ELMO1, EMB, KBTBD11, EML3, EOMES, FAM117A, FAM49A, FAM89B, FGF13, GAB3, GLIPR2, GMFG, GNPDA2, GRAMD4, HAAO, HEXB, ICAM2, IL10RA, ITGA4, ITGB1, ITGB2, KCNAB2, KCNN4, KLF2, KLF3, KLHL6, LEF1, LFNG, LPIN1, LYRM2, LYST, MPND, NCLN, PAQR7, PCED1B, PDE2A, PIK3R5, PODNL1, POGK, PRKCQ, PYHIN1, RACGAP1, RASA3, RASGRP2, RBM43, S1PR1, S1PR4, S1PR5, SAMHD1, SBK1, SETX, SH2D1A, SIDT1, SMPDL3B, SNX10, ST3GAL1, STK38, TBXA2R, TCF7, THAP7, TMEM71, TSR3, TTC7B, TXK, TXNDC5, VOPP1, XRN2, ACPL2, CD97, CMAH, FAM65B

Supplementary Table 4: Functional enrichment analysis of liver-resident CD56bright NK cells versus peripheral blood CD56bright NK cells

Source	Term Name	Term ID	Adjusted *P*-value
GO:BP	immune system process	GO:0002376	7.00E-23
GO:BP	leukocyte activation	GO:0045321	2.59E-16
GO:BP	immune response	GO:0006955	5.00E-16
GO:BP	cell activation	GO:0001775	1.46E-15
REAC	Cytokine Signaling in Immune system	REAC:R-HSA-1280215	7.40E-15
GO:BP	regulation of immune system process	GO:0002682	8.89E-15
GO:BP	immune effector process	GO:0002252	3.40E-14
GO:BP	T cell activation	GO:0042110	3.11E-13
GO:BP	regulation of leukocyte activation	GO:0002694	6.05E-12
GO:BP	lymphocyte activation	GO:0046649	1.08E-11
REAC	Interferon alpha/beta signaling	REAC:R-HSA-909733	1.32E-11
REAC	Immune System	REAC:R-HSA-168256	3.42E-11
GO:BP	type I interferon signaling pathway	GO:0060337	3.73E-11
GO:BP	cellular response to type I interferon	GO:0071357	3.73E-11
GO:BP	leukocyte differentiation	GO:0002521	4.59E-11
GO:BP	response to cytokine	GO:0034097	4.88E-11
GO:BP	regulation of cell activation	GO:0050865	5.21E-11
GO:BP	cytokine-mediated signaling pathway	GO:0019221	9.99E-11
GO:BP	response to type I interferon	GO:0034340	1.01E-10
GO:BP	alpha-beta T cell activation	GO:0046631	1.08E-10
GO:BP	regulation of T cell activation	GO:0050863	1.83E-10
GO:BP	response to biotic stimulus	GO:0009607	2.88E-10
GO:BP	response to other organism	GO:0051707	3.86E-10
GO:BP	response to external biotic stimulus	GO:0043207	4.20E-10
GO:BP	immune system development	GO:0002520	1.58E-09
GO:BP	hematopoietic or lymphoid organ development	GO:0048534	1.77E-09
GO:BP	cellular response to cytokine stimulus	GO:0071345	2.33E-09
GO:BP	positive regulation of immune system process	GO:0002684	3.52E-09
REAC	Interferon Signaling	REAC:R-HSA-913531	3.88E-09
GO:BP	hemopoiesis	GO:0030097	4.11E-09
GO:BP	leukocyte cell-cell adhesion	GO:0007159	4.32E-09
GO:BP	lymphocyte differentiation	GO:0030098	6.25E-09
GO:BP	regulation of leukocyte cell-cell adhesion	GO:1903037	9.19E-09
GO:BP	regulation of lymphocyte activation	GO:0051249	1.05E-08
GO:BP	response to external stimulus	GO:0009605	1.09E-08
GO:BP	response to virus	GO:0009615	1.52E-08
GO:BP	interspecies interaction between organisms	GO:0044419	3.08E-08

GO:BP	regulation of response to stimulus	GO:0048583	6.82E-08
GO:BP	biological adhesion	GO:0022610	7.26E-08
GO:BP	T cell differentiation	GO:0030217	9.81E-08
GO:BP	cell adhesion	GO:0007155	1.24E-07
GO:BP	regulation of cell adhesion	GO:0030155	1.62E-07
GO:BP	defense response to virus	GO:0051607	1.66E-07
GO:BP	defense response	GO:0006952	1.71E-07
GO:BP	regulation of leukocyte differentiation	GO:1902105	1.75E-07
GO:BP	positive regulation of leukocyte cell-cell adhesion	GO:1903039	2.22E-07
GO:BP	response to stress	GO:0006950	3.13E-07
GO:BP	regulation of multicellular organismal process	GO:0051239	4.22E-07
GO:BP	regulation of cell-cell adhesion	GO:0022407	4.26E-07
GO:BP	positive regulation of multicellular organismal process	GO:0051240	4.32E-07
GO:BP	cell surface receptor signaling pathway	GO:0007166	4.37E-07
GO:BP	positive regulation of T cell activation	GO:0050870	4.40E-07
GO:BP	positive regulation of cell activation	GO:0050867	4.65E-07
GO:BP	positive regulation of leukocyte activation	GO:0002696	5.04E-07
GO:BP	myeloid leukocyte activation	GO:0002274	5.07E-07
GO:BP	CD4-positive, alpha-beta T cell activation	GO:0035710	5.66E-07
GO:BP	positive regulation of cell adhesion	GO:0045785	6.75E-07
GO:BP	response to organic substance	GO:0010033	1.13201E-06
GO:BP	cellular response to organic substance	GO:0071310	1.39472E-06
GO:BP	response to interferon-alpha	GO:0035455	1.55613E-06
GO:BP	positive regulation of hemopoiesis	GO:1903708	1.90486E-06
GO:BP	alpha-beta T cell differentiation	GO:0046632	2.37984E-06
GO:BP	leukocyte proliferation	GO:0070661	2.43872E-06
GO:BP	regulation of intracellular signal transduction	GO:1902531	2.53022E-06
GO:BP	interferon-gamma production	GO:0032609	2.69463E-06
GO:BP	regulation of molecular function	GO:0065009	2.73685E-06
GO:BP	regulation of signaling	GO:0023051	3.33121E-06
GO:BP	cytokine production	GO:0001816	3.34291E-06
GO:BP	defense response to other organism	GO:0098542	3.7411E-06
GO:BP	viral life cycle	GO:0019058	4.17098E-06
GO:BP	positive regulation of leukocyte differentiation	GO:1902107	4.60413E-06
GO:BP	regulation of signal transduction	GO:0009966	5.91401E-06
GO:BP	intracellular signal transduction	GO:0035556	6.03729E-06
GO:BP	response to stimulus	GO:0050896	7.64358E-06
GO:BP	regulation of cytokine production	GO:0001817	7.89258E-06
GO:BP	cellular response to chemical stimulus	GO:0070887	8.01913E-06
GO:BP	apoptotic process	GO:0006915	8.78504E-06

GO:BP	negative regulation of immune system process	GO:0002683	8.79494E-06
GO:BP	regulation of interferon-gamma production	GO:0032649	1.2927E-05
GO:BP	positive regulation of cell-cell adhesion	GO:0022409	1.59678E-05
GO:BP	leukocyte activation involved in immune response	GO:0002366	1.70625E-05
GO:BP	positive regulation of cytokine production	GO:0001819	1.7758E-05
GO:BP	cell activation involved in immune response	GO:0002263	2.08217E-05
GO:BP	regulation of cell communication	GO:0010646	2.11051E-05
GO:BP	regulation of viral life cycle	GO:1903900	2.59607E-05
GO:BP	regulation of immune response	GO:0050776	2.72949E-05
GO:BP	cell-cell adhesion	GO:0098609	3.44019E-05
GO:BP	regulation of viral genome replication	GO:0045069	3.6024E-05
KEGG	Hepatitis C	KEGG:05160	3.66394E-05
GO:BP	regulation of leukocyte proliferation	GO:0070663	3.89028E-05
GO:BP	innate immune response	GO:0045087	4.14328E-05
GO:BP	regulation of catalytic activity	GO:0050790	5.65859E-05
GO:BP	positive regulation of lymphocyte activation	GO:0051251	5.8565E-05
GO:BP	regulation of hemopoiesis	GO:1903706	6.33616E-05
GO:BP	negative regulation of viral genome replication	GO:0045071	6.81515E-05
GO:BP	positive regulation of macromolecule metabolic process	GO:0010604	7.14836E-05
GO:BP	positive regulation of leukocyte proliferation	GO:0070665	7.71619E-05
GO:BP	cell death	GO:0008219	8.16649E-05
KEGG	Measles	KEGG:05162	8.24625E-05
REAC	Antiviral mechanism by IFN-stimulated genes	REAC:R-HSA-1169410	9.77261E-05
GO:BP	leukocyte mediated immunity	GO:0002443	9.88455E-05
GO:BP	regulation of cell death	GO:0010941	0.000114359
GO:BP	regulation of alpha-beta T cell activation	GO:0046634	0.000117476
GO:BP	positive regulation of CD4-positive, alpha-beta T cell proliferation	GO:2000563	0.000121728
GO:BP	regulation of cell killing	GO:0031341	0.000123569
GO:BP	positive regulation of metabolic process	GO:0009893	0.00012551
GO:BP	lymphocyte proliferation	GO:0046651	0.000129319
GO:BP	positive regulation of biological process	GO:0048518	0.000159803
GO:BP	mononuclear cell proliferation	GO:0032943	0.000163157
REAC	Signaling by Interleukins	REAC:R-HSA-449147	0.000169936
GO:BP	programmed cell death	GO:0012501	0.000248041
KEGG	Acute myeloid leukemia	KEGG:05221	0.000251813
GO:BP	viral genome replication	GO:0019079	0.000296892
GO:BP	response to interferon-gamma	GO:0034341	0.000313613
GO:BP	regulation of lymphocyte differentiation	GO:0045619	0.000324855
GO:BP	regulation of lymphocyte proliferation	GO:0050670	0.000326491
GO:BP	regulation of developmental process	GO:0050793	0.000333801

GO:BP	positive regulation of signaling	GO:0023056	0.000365836
GO:BP	regulation of mononuclear cell proliferation	GO:0032944	0.000388658
GO:BP	regulation of localization	GO:0032879	0.000393151
GO:BP	regulation of programmed cell death	GO:0043067	0.000450133
KEGG	Influenza A	KEGG:05164	0.000450237
GO:BP	MAPK cascade	GO:0000165	0.000500188
GO:BP	positive regulation of response to stimulus	GO:0048584	0.000599008
GO:BP	regulation of viral process	GO:0050792	0.000606025
GO:BP	positive regulation of developmental process	GO:0051094	0.000664419
GO:BP	signal transduction by protein phosphorylation	GO:0023014	0.000700954
GO:BP	regulation of apoptotic process	GO:0042981	0.000764887
GO:BP	regulation of MAPK cascade	GO:0043408	0.000789738
GO:BP	positive regulation of intracellular signal transduction	GO:1902533	0.000811215
REAC	Estrogen-dependent nuclear events downstream of ESR-membrane signaling	REAC:R-HSA-9634638	0.000850341
GO:BP	regulation of response to stress	GO:0080134	0.00087964
GO:BP	response to molecule of bacterial origin	GO:0002237	0.000882171
KEGG	Chemokine signaling pathway	KEGG:04062	0.000939578
GO:BP	positive regulation of cellular process	GO:0048522	0.000996043
GO:BP	positive regulation of cell communication	GO:0010647	0.00103145
GO:BP	signal transduction	GO:0007165	0.00106278
GO:BP	cell killing	GO:0001906	0.001128974
GO:BP	regulation of T cell differentiation	GO:0045580	0.001135157
GO:BP	regulation of cellular component movement	GO:0051270	0.001201697
GO:BP	negative regulation of viral life cycle	GO:1903901	0.001226156
GO:BP	positive regulation of protein phosphorylation	GO:0001934	0.001319011
GO:BP	leukocyte mediated cytotoxicity	GO:0001909	0.001388606
GO:BP	negative regulation of biological process	GO:0048519	0.001430091
GO:BP	positive regulation of protein kinase activity	GO:0045860	0.001453616
GO:BP	positive regulation of lymphocyte proliferation	GO:0050671	0.001531273
GO:BP	CD4-positive, alpha-beta T cell differentiation	GO:0043367	0.00158196
GO:BP	positive regulation of mononuclear cell proliferation	GO:0032946	0.001707031
GO:BP	positive regulation of cell population proliferation	GO:0008284	0.001732923
GO:BP	positive regulation of signal transduction	GO:0009967	0.001755233

This table has been truncated to the top 150 enriched terms selected on the basis of lowest adjusted *P*-value. GO:BP, Gene Ontology biological process database; REAC, Reactome pathway database; KEGG, KEGG pathway database.

3 Supplementary Figures

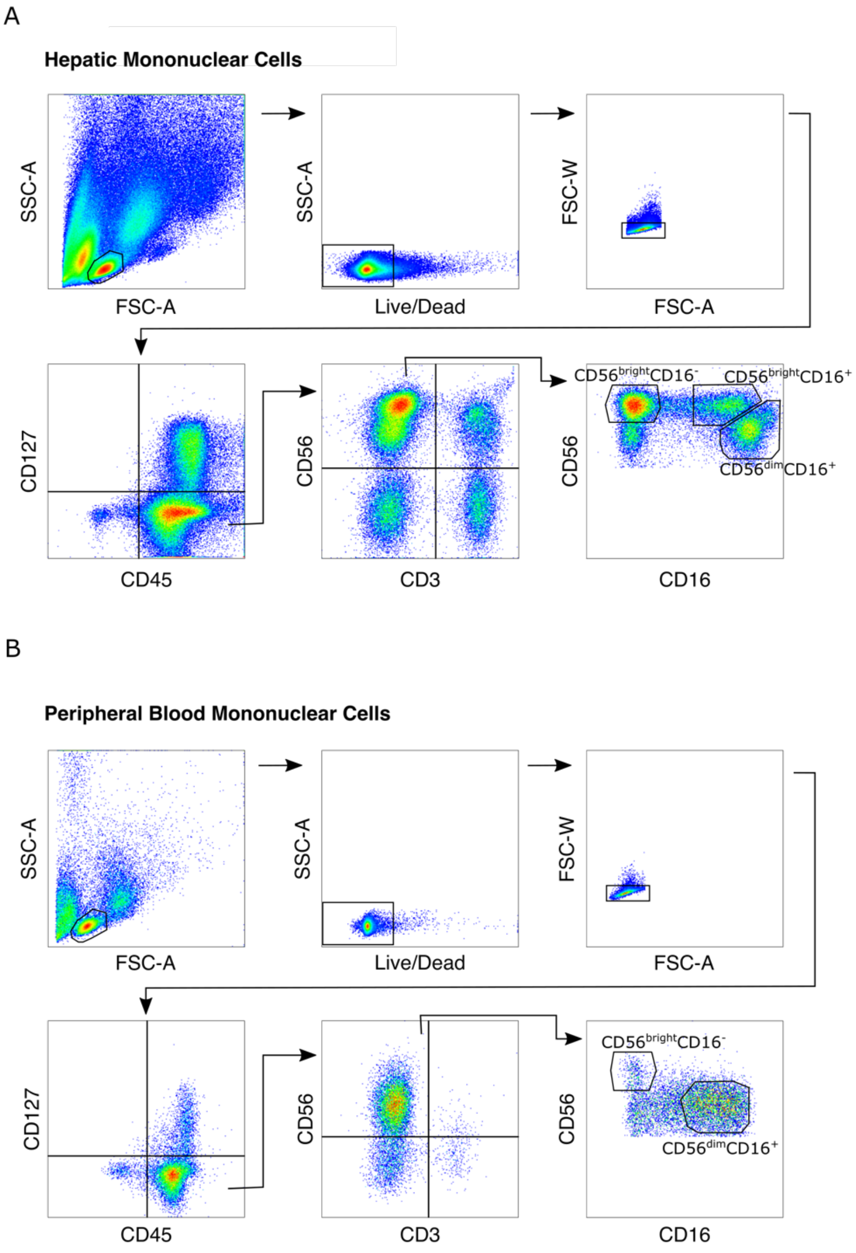

Supplementary Figure 1: Gating strategy for FACS analysis of NK cell populations

Flow cytometry gating strategy for RNA sequencing analysis of sorted NK cell subpopulations. **A,** Gating strategy for the analysis of NK cells derived from liver perfusate and tissue samples (HMNCs). **B,** Gating strategy for the analysis of NK cells derived from peripheral blood samples (PBMCs).

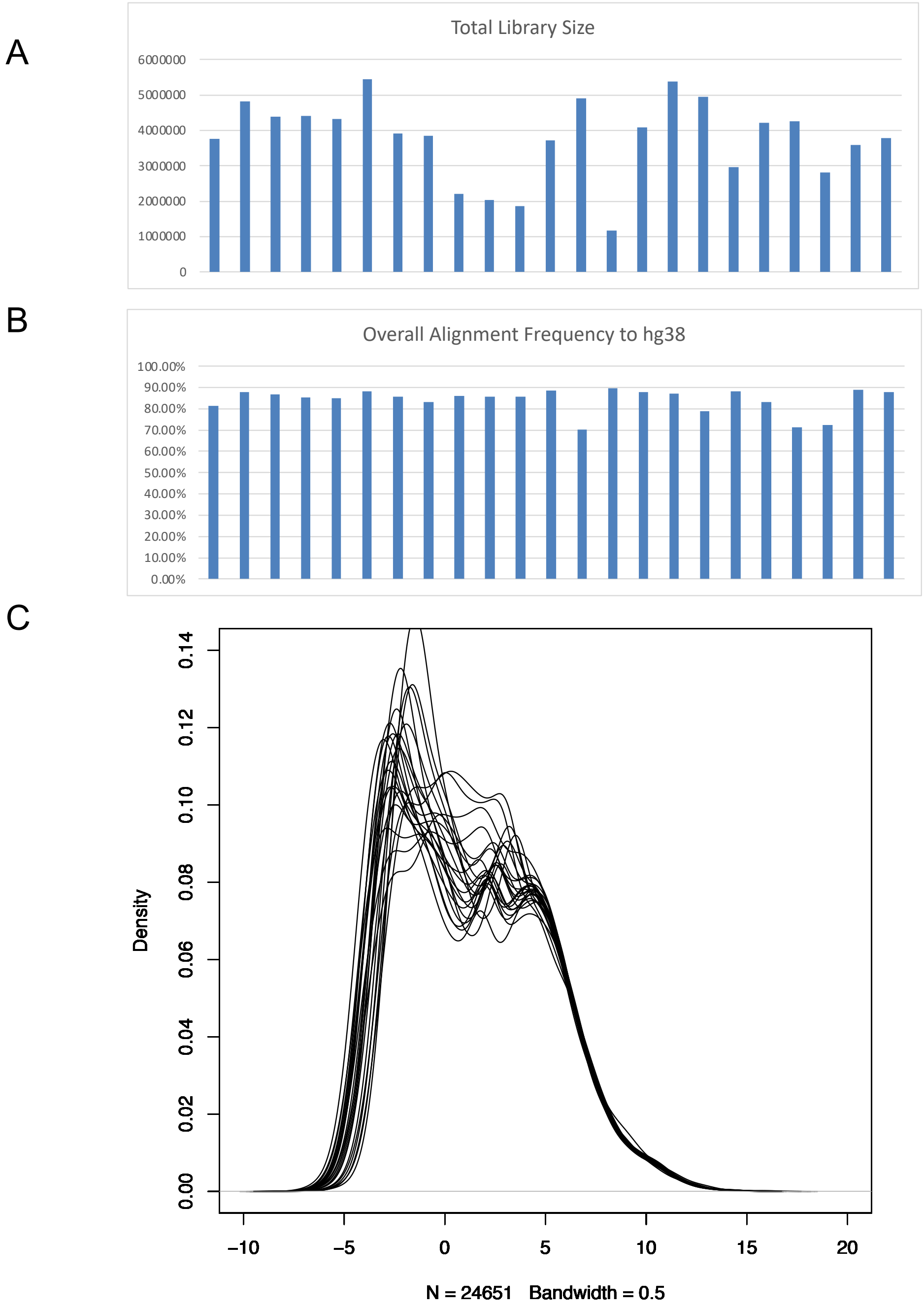

Supplementary Figure 2: RNA sequencing read counts and distribution

A, Total library size from the RNA sequencing libraries following data cleaning and filtering. **B,** The alignment frequency of total reads to hg38. **C,** Density plot showing the normalised counts for each sample.

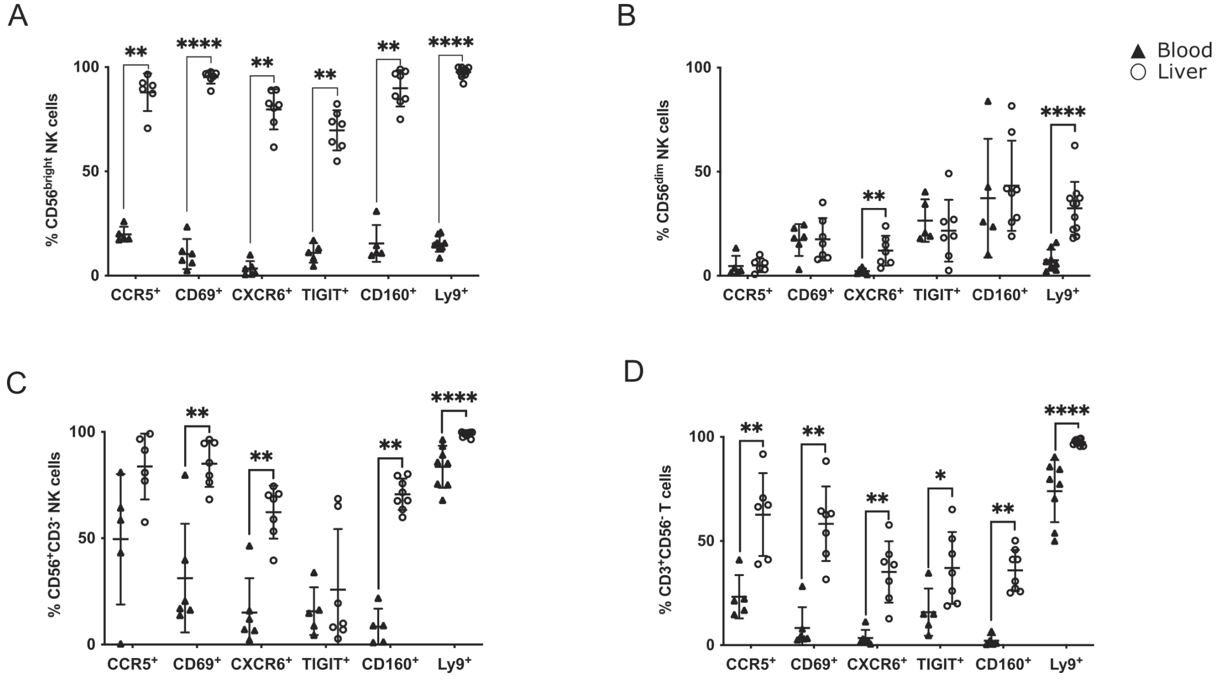

Supplementary Figure 3: Cell surface protein expression on NK and T cell sub-populations

Flow cytometry analysis of immune cells isolated from liver perfusate and peripheral blood samples (n5-8 blood, n=5-11 perfusate). Cells were stained with mAbs against CCR5, CD69, CXCR6, TIGIT, CD160 and Ly9. **A-D,** Percentage expression of each marker on $CD56^{bright}$ NK cells **(A)**, $CD56^{dim}$ NK cells **(B)**, $CD3^{+}CD56^{-}$ T cells **(C)** and $CD56^{+}CD3^{+}$ T cells **(D)** in blood (triangle) and liver perfusate (circle) samples. Data analysed using a Mann Whitney t test.*p<0.05, **p<0.01, ****p<0.0001

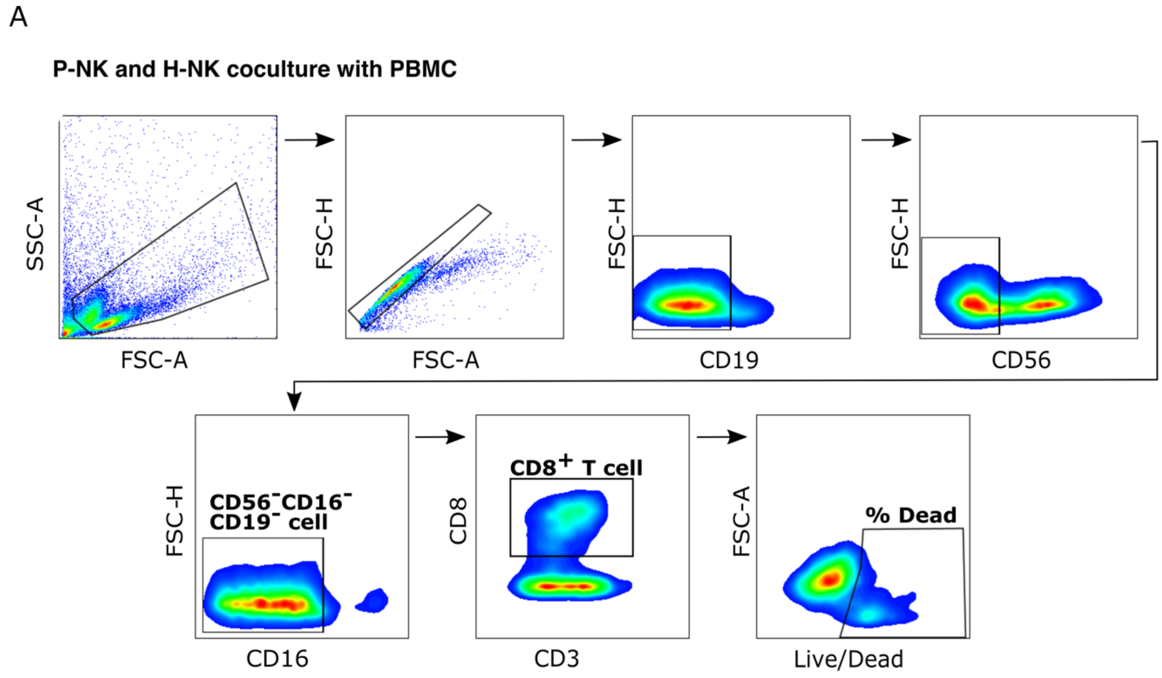

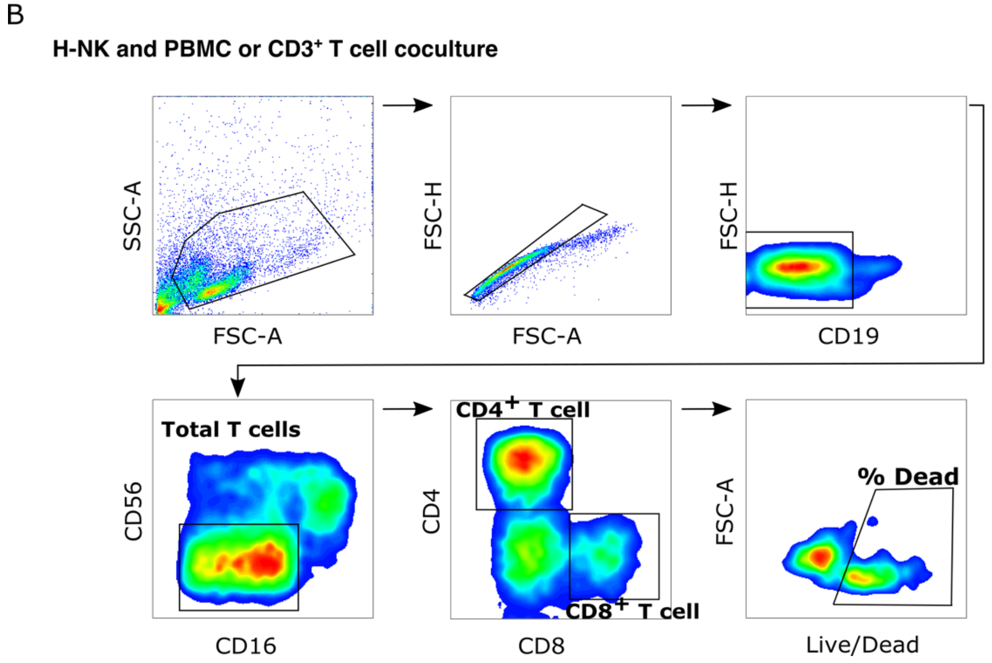

Supplementary Figure 4: Gating strategy for cytotoxicity experiments

Flow cytometry gating strategies for analysis of T cell death post coculture with NK cells for 24hours. **A,** Gating strategy for the analysis of T cells post coculture with peripheral blood NK (P-NK) cells or hepatic NK (H-NK) cells at varying ratios (corresponding to data in Figure 3). **B,** Gating strategy for the analysis of T cell populations post coculture with H-NK cells at a 1:1 ratio (corresponding to data in Figure 4).

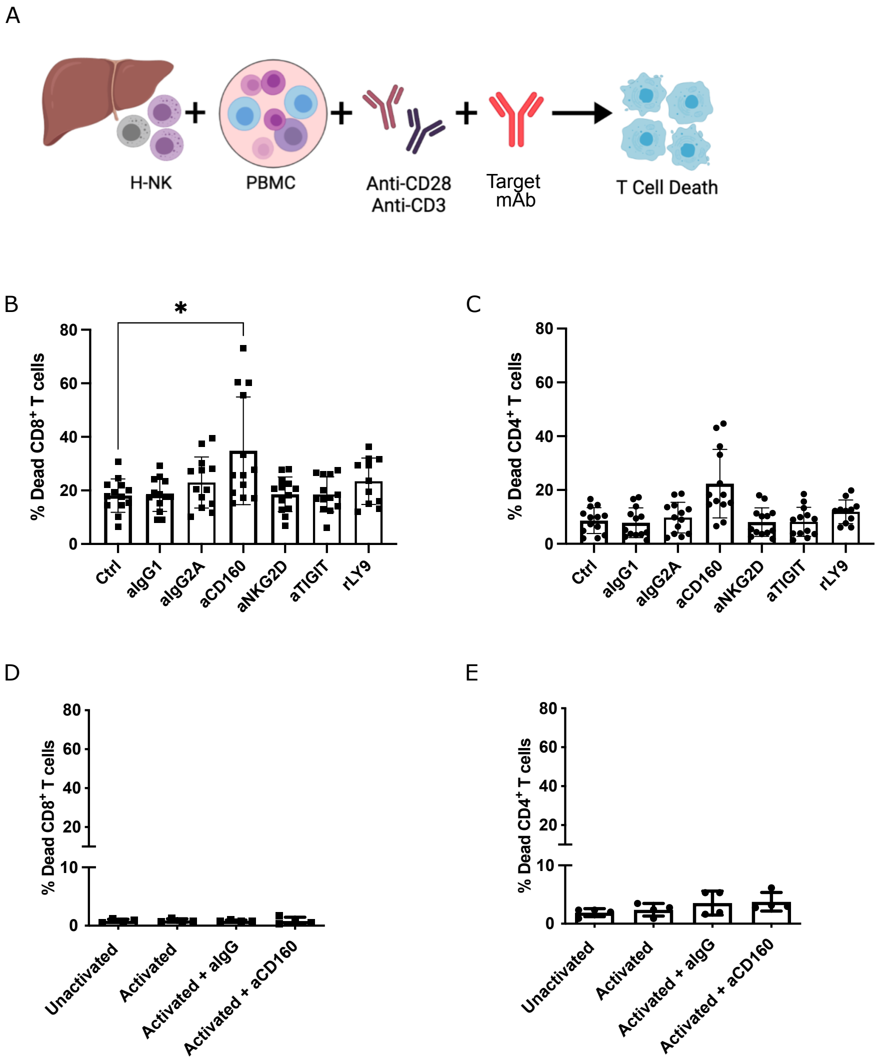

Supplementary Figure 5: Hepatic NK cells' killing of activated CD8+ T cells is enhanced via CD160 engagement and this is not via a direct effect of the anti-CD160 mAb on T cells.

PBMCs were activated with anti-CD3/28 and cultured alone or with H-NK cells and mAbs against CD160, NKG2D, ICAM-1, TIGIT, LFA-1, IgG1, IgG2A controls or recombinant Ly9 protein for 24 hrs (n=11-13). **A,** Schematic illustrating the experimental coculture set up. **B-C,** Percent dead CD8+ T cells **(B)** and CD4+ T cells **(C)** post-coculture with H-NK in activated control (Ctrl) and each treatment group. **D-E,** Percent dead CD8+ T cells **(D)** and CD4+ T cells **(E)** in PBMC monocultures in specified treatment groups. Data analysed by Friedman test with Dunn's multiple comparison test. *, $p<0.05$.
